# Hydrophilic Silica Nanoparticles in O/W Emulsion: Insights from Molecular Dynamics Simulation

**DOI:** 10.3390/molecules27238407

**Published:** 2022-12-01

**Authors:** Shasha Liu, Hengming Zhang, Shiling Yuan

**Affiliations:** 1 School of Chemistry and Chemical Engineering, Shandong University, Jinan 250100, China; 2 School of Chemistry and Chemical Engineering, Qilu Normal University, Jinan 250100, China

**Keywords:** silica nanoparticles, oil droplet, adsorption, coalescence, molecular simulations

## Abstract

Previous studies have been carried out on the effect of silica nanoparticles (SNPs) on the stability of oil–water emulsions. However, the combining configuration of SNPs and oil droplets at the molecular level and the effect of SNP content on the coalescence behavior of oil droplets cannot be obtained through experiments. In this paper, molecular dynamics (MD) simulation was performed to investigate the adsorption configuration of hydrophilic SNPs in an O/W emulsion system, and the effect of adsorption of SNPs on coalescence of oil droplets. The simulation results showed: (i) SNPs adsorbed on the surface of oil droplets, and excessive SNPs self-aggregated and connected by hydrogen bonds. (ii) Partially hydrophilic asphaltene and resin molecules formed adsorption configurations with SNPs, which changed the distribution of oil droplet components. Furthermore, compared with hydrophobic asphaltene, the hydrophilic asphaltene was easier to combine with SNPs. (iii) SNPs would extend the oil droplet coalescence time, and the π–π stacking structures were formed between asphaltene and asphaltene or resin molecules to enhance the connection between oil droplets during the oil droplet contact process. (iv) Enough SNPs tightly wrapped around the oil droplet, similar to the formation of a rigid film on the surface of an oil droplet, which hindered the contact and coalescence of components between oil droplets.

## 1. Introduction

Solid nanoparticles with appropriate surface wettability can stabilize emulsions, which retain the properties of traditional emulsions stabilized by surfactants [1,2]. In the production of unconventional petroleum such as oil extraction from oil/tar sands, oil shale and heavy petroleum reservoirs, the solid nanoparticles produced include silica, clay minerals in outputs [3,4] and fine carbonates [5,6]. These solids usually stabilize oil–water emulsions and upset the oil–water separation processes [7]. At the same time, solid nanoparticles may be carried downstream from the process, where they can cause serious damage to the process and equipment. Therefore, the study of solid nanoparticles in emulsion has become an increasingly important research topic in academia and industry [8].

At present, the stability of oil–water emulsions influenced by solid nanoparticles has been studied extensively. Researchers have found that reservoir particles (silica, clay and mineral scales) in petroleum fluids may be modified by adsorption of heavy crude oil components such as resin and asphaltene when they are in contact with crude oil [9,10]. Kralova et al. [11] carried out adsorption studies on particles and oil components by the QCM-D technique, and found the adsorbed amount from resin solution is dramatically smaller than when asphaltenes contribute to the adsorption. Subsequently, Kralova et al. used shear to study the effect of hydrophilic and hydrophobic silica particles on the rheological property of the oil–water emulsion system. It was found that the hydrophilic silica nanoparticles make the emulsion system have a gel property. Perino et al. [12] studied the effect of silica particles on water-in-crude oil emulsions by stability tests, interfacial tension measurements, dilatational rheology of the interface, rheology of the particle dispersions and cryo-SEM analysis. According to the different wettability of particles, the different mechanisms of emulsion stability in the presence of particles was discussed. Whatever the composition of the oil phase, it was evident that hydrophilic particles destabilize water-in-oil emulsions, whereas hydrophobic particles stabilize them.

In recent years, researchers found that surfactants may be combined with solid nanoparticles to stabilize oil and water emulsions [13,14,15,16]. Zhu et al. [13] used hydrophilic silica nanoparticles and low-concentration alkyl polyoxyethylene monododecyl ether (C12En) nonionic surfactants as emulsifiers to stabilize oil-in-water (O/W) emulsions, which were stable at room temperature but demulsified at high temperature. Worthen et al. [15] combined hydrophilic nanoparticles (NPs) (i.e., bare colloidal silica) with the weakly interacting zwitterionic surfactant octanamide propyl betaine to form O/W emulsions. It was found that NPs and surfactant work together to produce finer emulsions, which were more stable than NPs or surfactant alone. Silica nanoparticles have been extensively studied as emulsifiers, but the experiments mainly focused on the influence of silica nanoparticles on the stability of oil–water emulsion. The understanding of the role of nanoparticles at the oil–water interface at the micro level is lacking. The correct understanding of the structural information of the interface between nanoparticles and oil–water is of great significance to the demulsification of oil–water emulsions, and even oil–water separation.

Molecular dynamics (MD) simulation can provide microstructure information and dynamic properties at the molecular level, which cannot be observed in experiments [17,18]. Fan et al. [19] systematically investigated the performances of 3 nm-diameter silica nanoparticles with different surface chemistry at the decane–water interface by molecular dynamics simulations. Their results clearly put forward the influence of the surface chemical structure of nanoparticles on the three-phase contact angle, desorption energy, rotation relaxation of nanoparticles, etc. De Lara et al. [20] used molecular dynamics simulation to compare the stability difference of different functionalized silica nanoparticles at the multi-component brine/NP/oil three-phase interface. They found that aromatic molecules adsorbed on the surface of nanoparticles to form plaque-like domains, which affected the stability of the oil/saltwater/NP interface. To date, we have found that the behavior simulation of nanoparticles at the oil droplet–water interface is rare, and the composition of the oil phase is relatively simple, which has a certain gap with the actual oil droplet composition. Some studies have found that the hydrophilic particles tend to stabilize oil-in-water emulsions, whereas hydrophobic particles tend to stabilize water-in-oil emulsions [21,22,23,24]. Therefore, in our work, we constructed an O/W emulsion system composed of heavy oil droplets and water molecules, added spherical hydrophilic silica nanoparticles (SNPs) into the emulsion system and used MD to explore the adsorption configurations of SNPs at the oil droplet–water interface. Meanwhile, we simulated the coalescence behavior of emulsified oil droplets in the presence of SNPs. Our research contents can provide a reference for understanding the combining of silica nanoparticles with oil droplets and the effect of nanoparticles on the coalescence behavior of oil droplets.

## 2. Simulation Details

### 2.1. Simulation and Force Field

**Figure 1:**
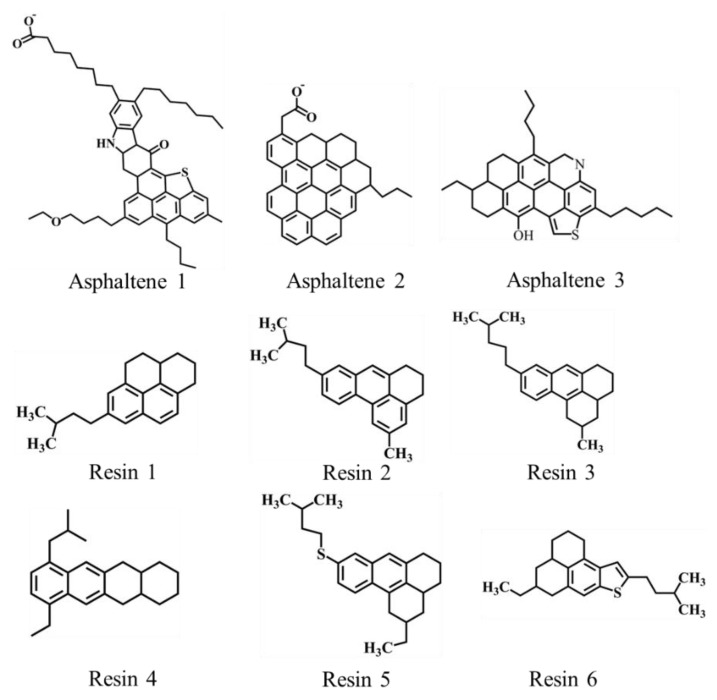
Asphaltene and resin molecules used in the simulations.

Our molecular dynamics simulation process was completed with the Gromacs2019 package, and the GROMOS 53a6 force field [25] was used. In all simulations, alkanes, cyclanes and aromatics were used as light oil components and asphaltenes and resins as heavy oil components. The types of hydrophilic and hydrophobic asphaltene and resin molecules were selected from our previous research work [18,26], as shown in Figure 1. Our SNP model was cut from silica crystal. The parameter sets of asphaltene, resins, hydrocarbon and SNPs were generated by the Automated Topology Builder (ATB) [27,28]. The water molecule was a simple point charge (SPC) model [29]. The parameters of sodium ions (Na^+^) that neutralize negative charge were referred to in [29].

A periodic boundary condition was applied along all dimensions in all simulations. The steepest descent method was used to minimize the energy in each system. In NVT and NPT ensembles, a velocity rescaling thermostat was used to maintain the system temperature at 300 K with a coupling constant of 0.1 ps. In the NPT ensemble, the ambient pressure was executed at 0.1 MPa by the Berendsen method with a coupling time constant of 1.0 ps for each system. The system compressibility was set as 4.5 × 10^−5^ bar^−1^. During our simulations, bond lengths were constrained by the LINCS algorithm. The van der Waals interaction used Lennard-Jones 12-6 potential, and the cutoff was set to 1.4 nm. Coulombic interaction used the particle-mesh Ewald (PME) summation method. Initial velocities were assigned according to Maxwell–Boltzmann distribution. The time step was 2 fs, and trajectory was saved every 10 ps.

### 2.2. Simulation Systems

#### 2.2.1. Emulsified Oil Droplet Model

In our simulation, light crude oil was composed of four types of alkanes, two types of cyclanes and two types of aromatics. The molecular models of light crude oil were based on Miranda et al.’s work [30,31], including alkanes (32 hexane, 29 heptane, 34 octane and 40 nonane molecules), cyclanes (22 cyclohexane and 35 cycloheptane molecules) and aromatics (13 benzene and 35 toluene molecules). The heavy crude oil components included two types of hydrophilic asphaltene [18], one type of hydrophobic asphaltene [26] (i.e., the number of each type of asphaltene is four) and six types of resins (i.e., the number of each type of resin is five). The concentration of resins and asphaltene in the crude oil was 38% of the weight of oil phase [26]. We used NPT and NVT ensembles to construct the O/W emulsion system. For specific build steps, please refer to the Supplementary Materials.

#### 2.2.2. Model of Emulsified Oil Droplet Containing SNPs

Different amounts of SNPs were randomly added to emulsion systems, and then MD was run to investigate the adsorption behavior of SNPs at the oil droplet–water interface. The compositions of emulsified oil droplet systems containing SNPs are shown in Table 1.

**Figure 2:**
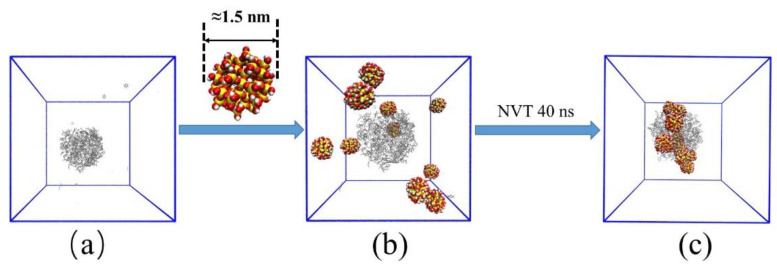
Schematic diagram of building emulsified oil droplet model with SNPs. For clarity, water molecules are not shown. Initial simulation box without SNPs (**a**). Initial simulation box with SNPs (**b**). Simulation box after NVT ensemble (**c**).

First, the above emulsified oil droplet was placed in the center of a new box (10 nm × 10 nm × 10 nm), and the SNPs were inserted into the box randomly (Figure 2b). Second, water molecules and the Na^+^ were added to it. Last, energy minimization simulation and 40 ns NVT ensemble simulation were carried out to obtain the equilibration of the emulsified oil droplet systems with SNPs (Figure 2c).

Destabilization and coalescence of droplets in emulsion are the only way to separate dispersed and continuous phases. Hence, the study of the specific kinetic behavior of oil droplets with SNPs during coalescence is particularly important for multiphase separation in emulsion systems. Here, we took the above emulsified oil droplets containing different amounts of SNPs as the research object to explore the influence of nanoparticles on oil droplet coalescence and the number of nanoparticles. The compositions of emulsion systems are shown in Table 2.

**Figure 3:**
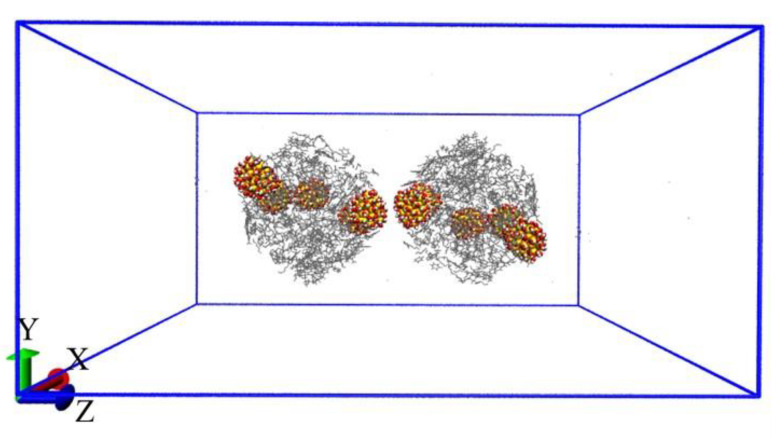
Lateral view of model simulation of oil droplets in O/W emulsion. For clarity, water molecules in the system are not shown.

We assumed that the oil droplets in emulsion had the following conditions: first, the centroids of two oil droplets are approximately along the z axis. In our simulations, two identical emulsified oil droplets were placed in the 10 nm × 10 nm × 20 nm box center, and the distance between two oil droplet centroids is about 7–9 nm, as shown in Figure 3. Then, the system was solvated by water molecules and Na^+^ ions. After the energy of each system was minimized, the NVT ensemble was run for 40 ns to simulate the demulsification process of oil droplets.

## 3. Results and Discussion

### 3.1. Adsorption Configuration of SNPs and Oil Droplet

**Figure 4:**
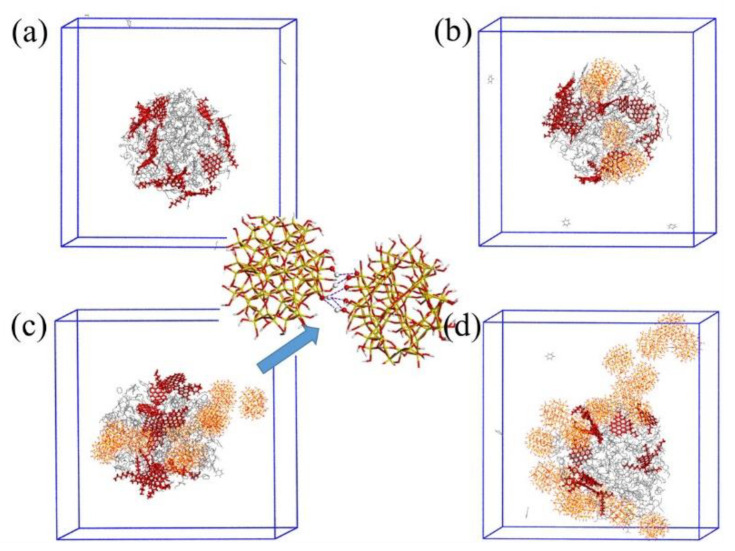
Snapshot of four systems in the last frame of NVT simulation. (**a**) System E-I without SNPs, (**b**) System E-II with five SNPs, (**c**) System E-III with ten SNPs, (**d**) System E-IV with twenty SNPs. In order to maintain the integrity of oil droplets, we placed the oil droplets in the center of boxes.

Different amounts of SNPs were added to the O/W systems for the 40 ns NVT ensemble, which was used to simulate the combination of the oil droplet and SNPs in the emulsion system. We extracted the oil droplet configurations of the last frame of the NVT ensemble of all systems, as shown in Figure 4. Asphaltene molecules are represented by the red rod models, resin, hydrocarbon molecules are represented by the gray linear models and SNPs are represented by the linear models. According to Figure 4a, asphaltene molecules were more uniformly distributed at the oil–water interface when SNPs were not added. After adding SNPs, the distribution of asphaltene molecules changed, and SNPs migrated from the dispersed state in the initial configuration to the oil–water interface, and finally adsorbed on the surface of oil droplets, as shown in Figure 4b–d. Moreover, with the increase in the number of SNPs, the stacking and self-aggregation of SNPs appeared on the surface of the oil droplet, which was similar to the previous theoretical study on the self-assembly behavior of silica nanoparticles at the water–decane interface [32]. The local enlarged view of stacked SNPs in Figure 4c shows that hydrogen bonds were formed between the −OH groups at the contact interface of stacked SNPs, which made the interaction between SNPs more firm. At the same time, the distribution change of asphaltene molecules on the oil droplet surface also indicated that SNPs adsorbed on the oil droplet surface affect the distribution of oil droplet components, which is discussed below.

**Figure 5:**
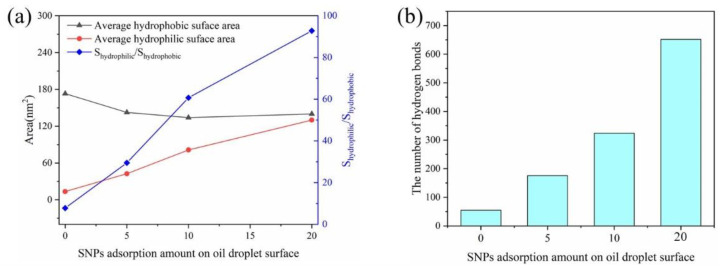
Average hydrophobic and hydrophilic surface areas of emulsified oil droplets (**a**). The number of hydrogen bonds formed between oil droplet and water molecules (**b**).

Due to the hydrophilicity of SNPs, we studied the surface condition of oil droplets with different SNP contents in each system, the solvent-accessible surface area (SASA) was calculated, as shown in Figure 5a, and the number of hydrogen bonds formed between oil droplet and water molecules is shown in Figure 5b. We carried out the calculations of SASA using the probe of 1.4 angstrom in the Gromacs2019 package. These results showed that with the increase in SNPs on the oil droplet surface, the hydrophilic surface area of the oil droplet increased, and the hydrophobic surface area decreased. In addition, with the increase in SNPs on the oil droplet surface, the number of hydrogen bonds increased simultaneously. It could be seen that the addition of hydrophilic SNPs to the system could change the surface properties of oil droplets, and the hydrophilicity was significantly enhanced.

Based on the experimental research and the influence of the above SNPs on the distribution of oil droplet components, we used local enlarged views, radial distribution function (RDF) and radial number density to deeply explore the distribution of asphaltene and resin molecules in oil droplets for System E-I, System E-II, System E-III and System E-IV.

**Figure 6:**
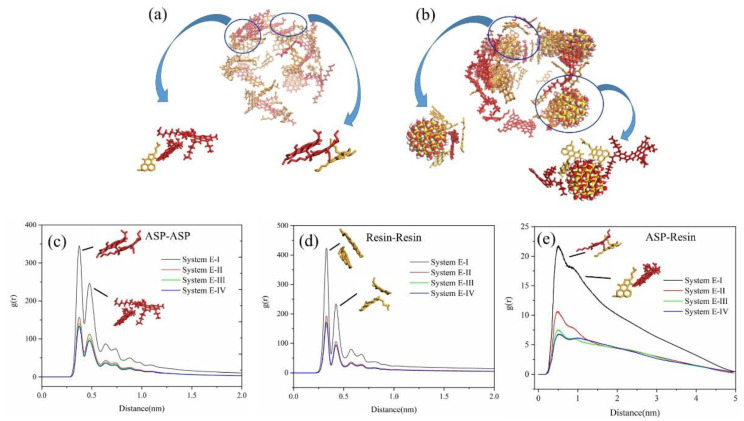
Distribution configurations of asphaltene, resin molecules and SNPs in System E-I (**a**) and System E-II (**b**). Radial distribution function (RDF) of four systems: asphaltene and asphaltene molecules to centroid (**c**), resin to resin (**d**), asphaltene to resin (**e**). Red, orange sticks represent asphaltene and resin molecules, respectively, spheres represent SNPs.

Taking the System E-I and System E-II as examples, we extracted the distribution configuration of asphaltene and resin molecules in System E-I in the last frame of NVT as shown in Figure 6a, and the distribution configuration of asphaltene, resin molecules and SNPs in System E-II in the last frame of NVT as shown in Figure 6b. Meanwhile, we show the relationship between asphaltene and resin molecules, as well as the relationship between SNPs and asphaltene and resin molecules by local enlarged views. Red molecules are asphaltene, yellow molecules are resin and cyan spheres are SNPs. From the results shown in Figure 6, it was evident that asphaltene molecules on the surface of oil droplets could form combination configurations of face-to-face stacking and edge-to-face stacking with resin molecules, when the system was free of SNPs. However, by adding SNPs into the system, the distribution of components in the oil droplets changes, and some asphaltene and resin molecules adsorbed around SNPs and combined with SNPs to form adsorption configurations.

The interactions between asphaltene and resin play a vital role in linking other components of oil droplets into an indivisible whole. Therefore, we calculated the RDF of asphaltene molecules, resin molecules and between asphaltene and resin molecules of the four systems in the last 5.0 ns of NVT to illustrate the stacking between asphaltene and resin as shown in Figure 6c–e. Each curve has two peaks in Figure 6c–e, and each peak represents a different aggregation mode between molecules. The first peaks correspond to the face-to-face stacking structure, and the second peaks correspond to the edge-to-face stacking structure. In Figure 6c–e, we noticed that the peak value of the face-to-face stacking structure of asphaltene or resin molecules was higher than that of edge-to-face, which indicated that face-to-face stacking structures were the main stacking structure among asphaltene or resin molecules. Moreover, it was evident that the peaks of face-to-face stacking structures and edge-to-face stacking structures for System E-I were higher than System E-II to E-IV, and with the increase in the number of SNPs adsorbed on the oil droplet surface, the peaks of face-to-face and face-to-face stacking structures of asphaltene, resin molecules and between asphaltene and resin molecules slowly decreased. It could be concluded that SNPs affect the distribution of asphaltene and resin molecules in oil droplets, which was mainly related to the adsorption of asphaltene and resin molecules on the SNP surface.

**Figure 7:**
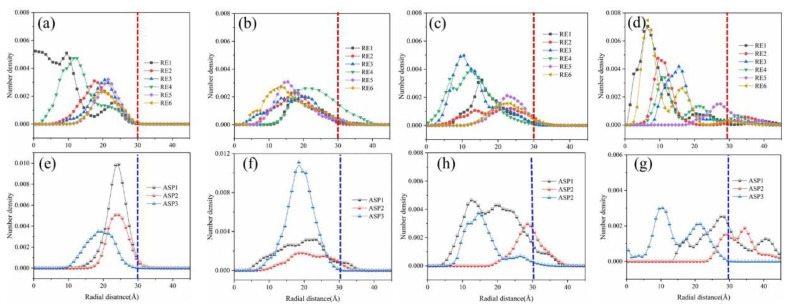
Radial number density of asphaltene and resin in the last 2.0 ns of NVT simulation for System E-I to System E-IV. The radial number density of six resin molecules (**a**–**d**). The radial number density of asphaltene molecules (**e**–**g**).

In order to further explore the influence of SNPs on the distribution of asphaltene and resin molecules in oil droplets, we counted the radial number density of asphaltene and resin molecules in the last 2.0 ns of NVT simulation for four systems (Figure 7). The pictures of Figure 7a–d represent the radial number density statistics of six resin molecules, and the pictures of Figure 7e–g represent the radial number density statistics of three asphaltene molecules. In Figure 7a, hydrophobic asphaltene and resin molecules were distributed in the range of the oil droplet with radius of 3.0 nm, and hydrophilic asphaltenes (ASP1 and ASP2) were close to the edge of the oil droplet. However, some resin and asphaltene molecules extended beyond 3.0 nm after the addition of SNPs to systems as shown in Figure 7b–d. These show that some resin molecules were distributed from the inside of the oil droplet to the edge. Furthermore, in contrast to the three types of asphaltene, the hydrophilic asphaltenes were always close to the oil droplet edge, even in the presence of SNPs. It was also indicated that SNPs were easier to combine with hydrophilic asphaltene. Changes in the distribution of resin and asphaltene molecules were related to their formation of combining configurations with SNPs. The statistical results of the radial number density of asphaltene and resin of the four systems proved again that the adsorption of SNPs on the surface of oil droplets would affect the distribution of oil droplet components. Some resin molecules appeared the oil droplet edge, and hydrophilic asphaltene molecules were more easily combined with silica nanoparticles.

### 3.2. Effects of SNPs on the Coalescence Behavior of Oil Droplets

#### 3.2.1. Oil Droplet Coalescence Process

**Figure 8:**
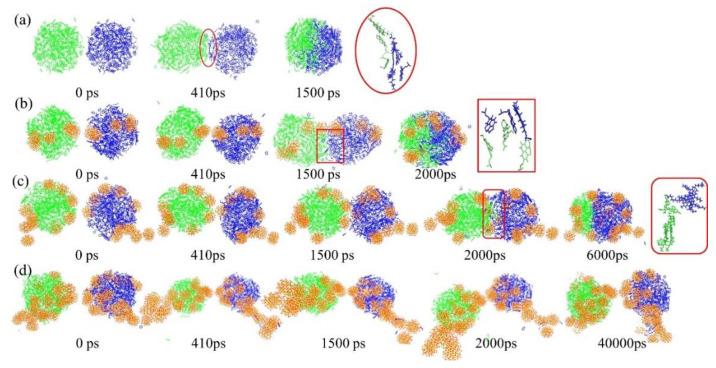
Snapshots of droplet coalescence. In order to distinguish the two droplet components, the two droplet components are represented by green and blue linear models. The coalescence behavior of oil droplets in System C-I (**a**). The coalescence behavior of oil droplets in System C-II (**b**). The coalescence behavior of oil droplets in System C-III (**c**). The coalescence behavior of oil droplets in System C-IV (**d**).

Figure 8 shows the distribution configurations of the two oil droplets at the same time during their coalescence process of System C-I, System C-II, System C-III and System C-IV. It can be seen from Figure 8 that the coalescence behavior of oil droplets in the four investigated systems was significantly different under the influence of silica nanoparticles. Moreover, it is worth noting that when the oil droplets adsorbed twenty SNPs in System C-IV, the two oil droplets could not completely coalescence, as shown in Figure 8d. Meanwhile, we used the local enlarged views to explore the combining configuration of oil droplet components at the oil droplet interface. From the local enlarged views, we discovered that in Figure 8a–c, π–π stacking structures were formed between the asphaltene or resin molecules at the interface of the two oil droplets, which enhanced the connection between oil droplet components. Figure S2 shows the change curve of the centroid distance between the two oil droplets with time. It can be observed that the centroid distance between the two oil droplets without SNPs decreased the fastest. Meanwhile, with the increase in the number SNPs adsorbed on the oil droplet surface, the coalescence time of oil droplets increased. For System C-IV, the centroid distance of oil droplets was almost unchanged. Therefore, the more SNPs on the oil droplet surface, the less conducive to coalescence of oil droplets.

**Figure 9:**
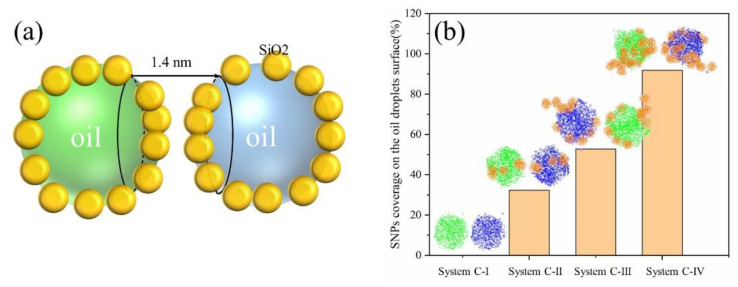
Schematic of SNPs within 1.4 nm of the adjacent interface between two oil droplets (**a**). The coverage of SNPs on the surface of oil droplets for four systems (**b**).

Considering different types of oil droplets in the emulsion system leads to different nanoparticle coverage on the interface of adjacent oil droplets, thus affecting the oil droplet coalescence time. We took the SNPs within 1.4 nm of the adjacent interface between two oil droplets as the calculated object (Figure 9a). The coverage rate of oil droplets was calculated using the SASA method, and the calculation formula is as follows:

$$\tau =\frac{S_{SNPs}+S_{oil}-S_{SNPs}{}_{+oil}}{2\cdot S_{SNPs}{}_{+oil}}$$

*S_SNPs_* is the area of silica nanoparticles on the surface of adjacent oil droplets. *S_oil_* represents the area of the oil components on the surface of adjacent oil droplets. *S_SNPs+oil_* is the total area of the surface of adjacent oil droplets. *τ* is the coverage rate of silica nanoparticles on the surface of adjacent oil droplets.

The results of coverage of SNPs at the interface of adjacent oil droplets are shown in Figure 9b. The coverage of SNPs at the oil droplet interface gradually increased from System C-I to System C-IV, and coverage of SNPs in System C-IV reached 91.8% as shown in Figure 9b. Therefore, we believed that when the SNP coverage on the oil droplet surface is greater than 92%, the emulsion system will always be difficult to demulsify. In conclusion, the more silica nanoparticles on the oil droplet surface, the longer the coalescence time of the oil droplets. When the surface of the oil droplet was coated with a large number of SNPs, the components of the two oil droplets could not establish effective contact and coalescence.

**Figure 10:**
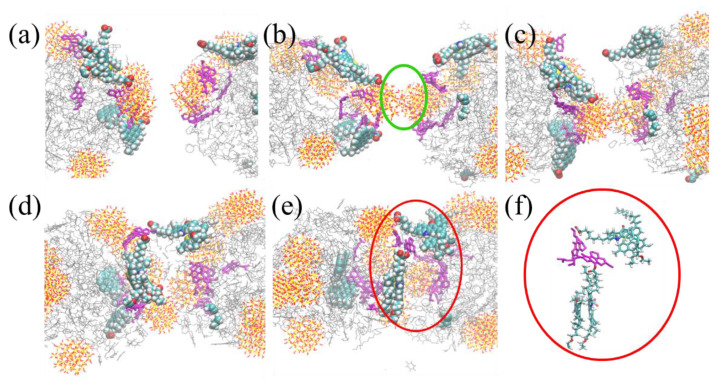
Oil droplet with five SNPs coalescence process. Configurational snapshot of the two oil droplets at 0 ps (**a**). Configurational snapshot of the two oil droplets at 450 ps, and the SNPs between the oil droplet interface contacted firstly in green circle (**b**). Configurational snapshot of the two oil droplets at 800 ps (**c**). Configurational snapshot of the two oil droplets at 1200 ps (**d**). Configurational snapshot of the two oil droplets at 1600 ps (**e**), and enlarged view of the interface at 1600 ps (**f**). Spherical models represent asphaltene molecules, and pink rod models represent resin molecules. Other components of oil droplet and SNPs are shown as line models.

In order to explore the behavior changes of oil droplet components during the coalescence process, we took System C-II with five SNPs as an example, and extracted the configurational snapshots of the two oil droplets at five different times. The interfacial asphaltene molecules are shown as spherical models, resins are shown as pink rod models and other components of the oil droplet and SNPs are shown as line models. We found that the SNPs between the oil droplet interface contacted first, as shown in Figure 10b. Then, the hydrophilic asphaltene molecules on the surface of the two oil droplets gradually extended into the solution as shown in Figure 10c,d. Finally, at the oil droplet interface, π–π stacking configurations were formed between the asphaltene molecules and between the asphaltene and resin molecules as shown in Figure 10e,f. This combining configuration made the two oil droplets connect more closely. These results suggested that when there were SNPs at the adjacent interface of the two oil droplets, the SNPs contacted first, and then the components of the oil droplets coalesced, so the nanoparticles would extend the coalescence time of the oil droplets. However, when the oil droplets are tightly wrapped by nanoparticles, a rigid film is formed on the surface of the oil droplets, which is not conducive to the contact and coalescence of components between oil droplets, and thus the demulsification of oil droplets cannot be realized.

#### 3.2.2. Steered Molecular Dynamics

**Figure 11:**
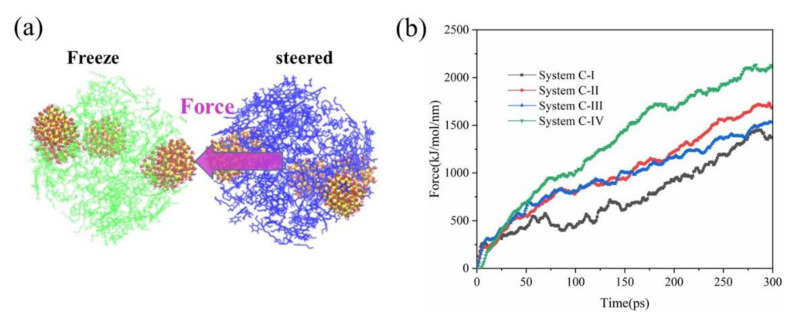
Schematic of the steered structure (**a**), force parameters during the steering (**b**).

In order to prove that effect of SNP addition on the mechanical properties of the oil droplet, steered molecular dynamics simulation was used. The change of force parameters could directly reflect mechanical properties of oil droplets. We used the grouping method to define a fixed group and a pulled group in the two oil droplets (Figure 11a), and applied a z-direction force to the pulled group. We use the harmonic potential for pulling. The pull force constant was set to 1000 kJ·mol^−1^·nm^−2^. The movement speed of the pulling group was 0.01 nm·ps^−1^, and the reference position of the pulled groups was the center of the fixed oil droplet. Taking the initial model of emulsion with two oil droplets as the initial steered molecular dynamics model, the change of the force parameters with time during the contact process of the two oil droplets under the action of external force is shown in Figure 11b. Obviously, in line with what we have observed, when there were no SNPs, the pulling force required for the oil droplet contact process was smaller; when the oil droplet surface adsorbed SNPs, the pulling force required for the oil droplet contact process increased. When the content of nanoparticles on the surface of the oil droplet was small, such as System C-II and System C-III, the pulling force required for the oil droplet coalescence was low, and the value of the pulling force had little difference during the oil droplet contact process. However, it is worth noting that the pulling force in System C-IV was significantly higher than that of other systems. Therefore, we obtained that SNPs hinder oil droplet coalescence, and when there are enough silica nanoparticles on the surface of oil droplets, it is difficult for oil droplets to coalesce.

## 4. Conclusions

Molecular dynamics simulation was used to study the adsorption configuration of silica nanoparticles and oil droplets in the O/W emulsion system. At the same time, the effect of the adsorption amount of silica nanoparticles on the surface of oil droplets on coalescence of oil droplets was investigated.

Firstly, the SNPs adsorbed on the surface of oil droplets, while too many SNPs self-aggregated on the surface of oil droplets, and they were connected by hydrogen bonds. At the same time, the SNPs form combining configurations with asphaltene and resin molecules. Secondly, by analyzing the RDF and radial number density of oil droplet asphaltene and resin, we found that the adsorption of SNPs on the surface of oil droplets would affect the distribution of oil droplet components. Some resin molecules appeared at the oil droplet edge, and hydrophilic asphaltene molecules were more easily combined with SNPs.

Exploring the effect of the adsorption amount of SNPs on coalescence of oil droplets, we discovered that the more SNPs on the surface of oil droplets, the longer the time required for oil droplet coalescence. However, when the SNP coverage on the oil droplet surface was greater than 92%, the components of the oil droplet had difficulty contacting and coalescing. We thought that when the oil droplets are tightly wrapped by nanoparticles, a rigid film would be formed on the surface of the oil droplets, which is not conducive to demulsification of oil droplets. Meanwhile, the π–π stacking structures were formed between asphaltene and asphaltene or resin molecules at the oil droplet interface, which enhanced the connection between oil droplets. Finally, the steered molecular dynamics showed that the more SNPs on the oil droplet surface, the greater the external force required during the contact process of two oil droplets. Therefore, we are more certain that the more nanoparticles adsorbed on the surface of the oil droplet, the less likely oil droplet demulsification. The above findings provide a reference for understanding the combining of silica nanoparticles with oil droplets and the effect of nanoparticles on the coalescence behavior of oil droplets.

## Figures and Tables

**Table 1:** Details of the emulsified oil droplet systems containing SNPs.

**System**	**Number of Molecules**			
	**Crude Oil Droplet**	**SNPs**	**Na** **^+^**	**Water**
E-I	1	0	8	30,806
E-II	1	5	8	29,985
E-III	1	10	8	29,260
E-IV	1	20	8	28,310

**Table 2:** Details of the emulsification systems for investigating the influence of SNPs on oil droplet coalescence.

System	Oil Droplet Type	Number of Molecules	
		Oil Droplet	Water
C-I	with zero SNPs	2	61,608
C-II	with five SNPs	2	60,829
C-III	with ten SNPs	2	58,868
C-IV	with twenty SNPs	2	28,310
			58,095

## Data Availability

Not applicable.

## Supplementary Materials

The following supporting information can be downloaded at: https://www.mdpi.com/article/10.3390/molecules27238407/s1, Figure S1: Schematic diagram of building emulsified oil droplet model; Figure S2: Variation of centroid distance between two oil droplets in four systems.
