# Supplementary material for: Hydrophilic Silica Nanoparticles in O/W Emulsion: Insights from Molecular Dynamics Simulation
Source: Molecules. 2022 Dec 1;27(23):8407. doi: 10.3390/molecules27238407 (PMC9740303; doi:10.3390/molecules27238407)
Supplement: Supplementary file 1 [file molecules-27-08407-s001.zip › molecules-2044386-supplementary.pdf]

Supporting information

Hydrophilic Silica Nanoparticles in O/W Emulsion: Insights from Molecular Dynamics Simulation

Shasha Liu[1,2], Hengming Zhang, Shiling Yuan[1,*]

[1]School of Chemistry and Chemical Engineering，Shandong University，Jinan 250100，China

[2]School of Chemistry and Chemical Engineering，Qilu Normal University，Jinan 250100，China

[*]Author to whom correspondence should be addressed, E-mail: shilingyuan@sdu.edu.cn

2.2.1 Emulsified oil droplet model

The specific emulsified oil droplet model building steps are as follows:

First, the components of crude oil including alkanes, cyclanes, aromatics, asphaltenes, and resins were randomly inserted into a cubic box (x = 10 nm, y = 10 nm, z = 10 nm), as shown in Figure S1a. Energy minimization was then performed for eliminating overlapping. After that, performing a 30 ns NPT ensemble simulation in order to obtain the reasonable density. The equilibrium configuration after NPT run was shown in Figure S1b. Second, the above crude oil was solvated with 19230 water molecules in an 8 nm × 8 nm ×8 nm simulation box (Figure S1c). Then, we carried out Energy minimization and a 20ns NVT ensemble MD simulation to obtain the emulsified oil droplet, as shown in Figure S1d.

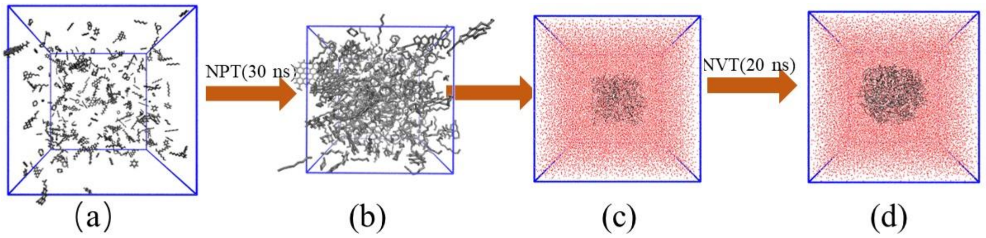

Figure S1. Schematic diagram of building emulsified oil droplet model. Initial simulation box with all oil molecules (a). Simulation box after NPT ensemble (b). Oil molecules are added to the simulation box and solvated by water molecules (c). Simulation box after NVT ensemble (d).

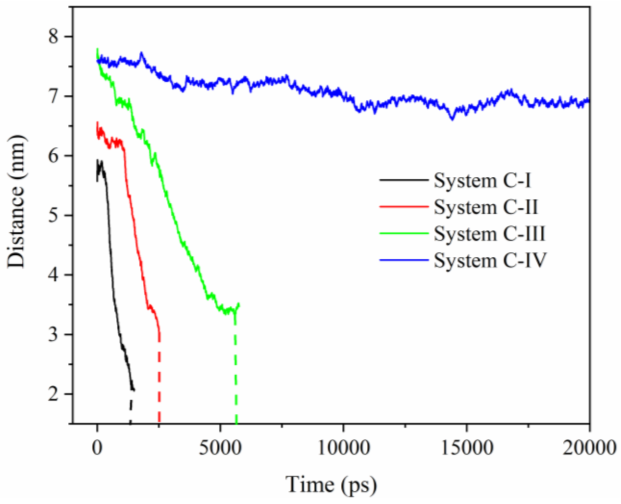

Figure S2. Variation of centroid distance between two oil droplets during coalescence process.
